# Supplementary material for: Estimating physical conditions supporting gradients of ATP concentration in the eukaryotic cell
Source: Biophys J. 2025 Jun 16;125(2):377–86. doi: 10.1016/j.bpj.2025.06.016 (PMC12968834; doi:10.1016/j.bpj.2025.06.016)
Supplement: Document S1. Figures S1–S6 [file mmc1.pdf]

Biophysical Journal, Volume *125*

Supplemental information

Estimating physical conditions supporting gradients of ATP concentration in the eukaryotic cell

Rajneesh Kumar and Iain G. Johnston

Supplementary Information

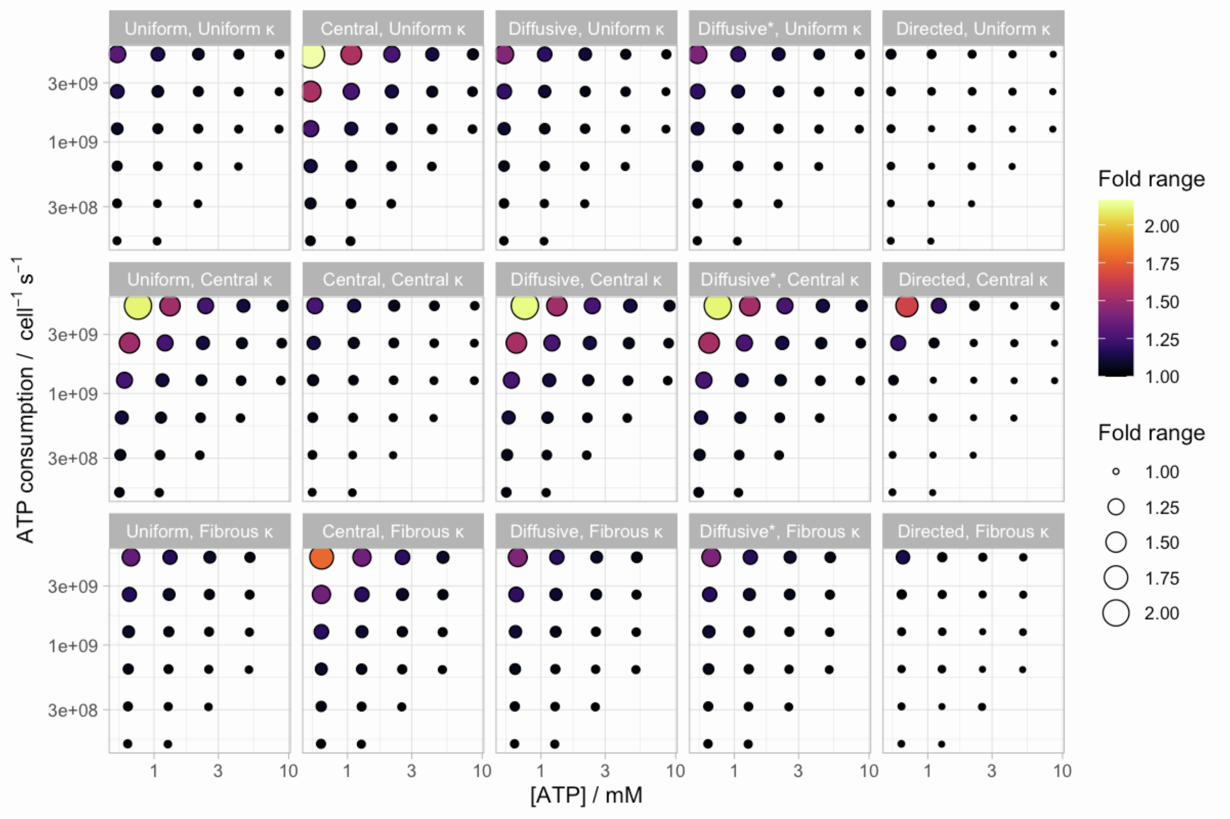

Supplementary Figure 1. **Fold-change in ATP concentration.** ATP concentration gradients plotted as fold change between minimum and maximum values in the cell, rather than CV as in the main text.

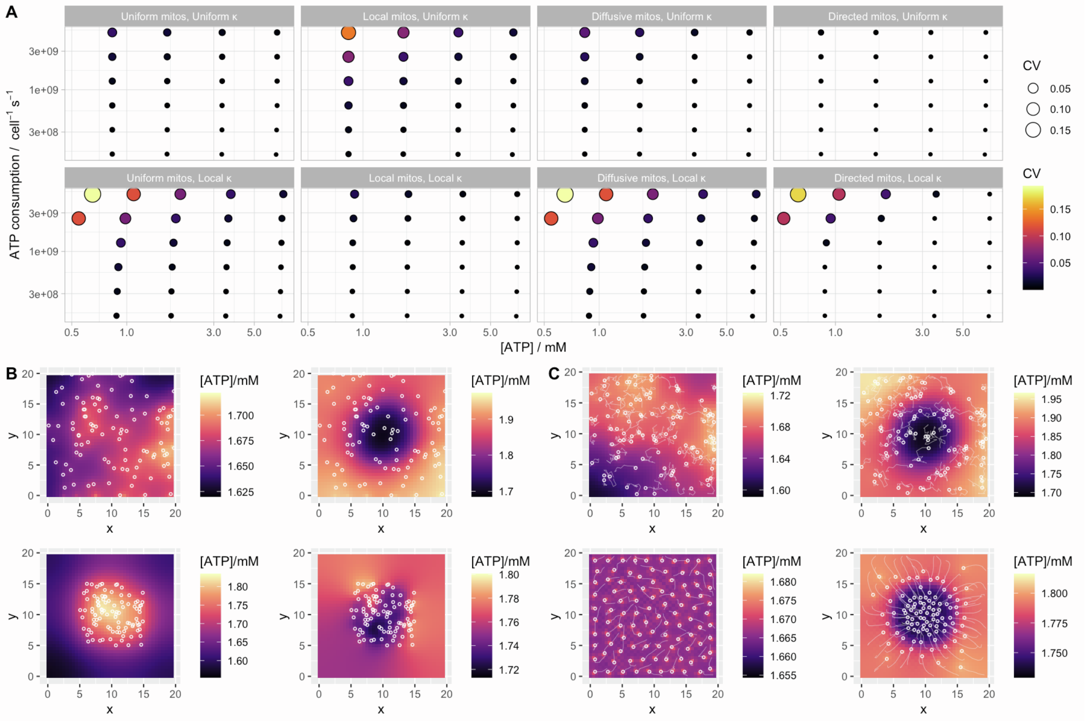

Supplementary Figure 2. **Smaller cell version of the model.** This approach uses a model cell 20 μm × 20 μm × 10 μm (hence volume 4 × 10^3 μm^3). (A) CV of ATP concentration as in Fig. 3. (B) Long-term concentration profile with static mitochondria as in Fig. 2. (C) Long-term concentration profile with static mitochondria as in Fig. 4. Parameters in (B-C) correspond to cellular consumption rates around 2.6 × 10^9 molecules s^{-1}.

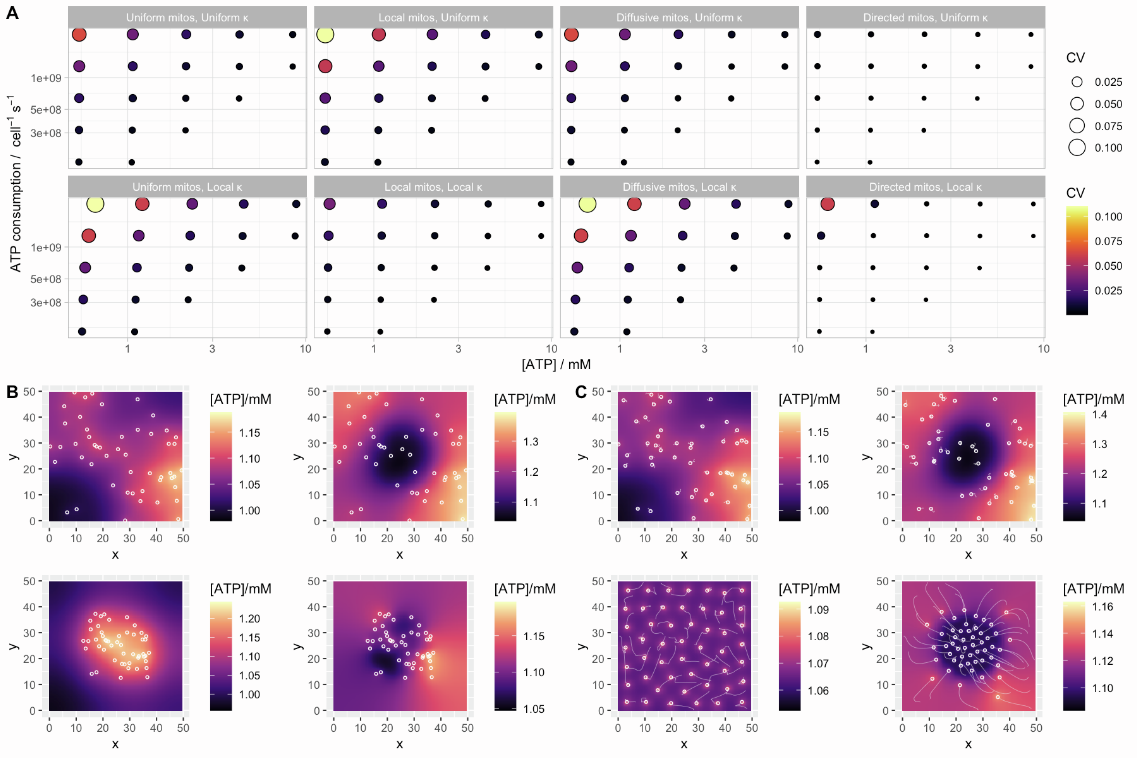

Supplementary Figure 3. **Model with fewer mitochondria.** 50 mitochondria in the simulated cell. (A) CV of ATP concentration as in Fig. 3. (B) Long-term concentration profile with static mitochondria as in Fig. 2. (C) Long-term concentration profile with static mitochondria as in Fig. 4.

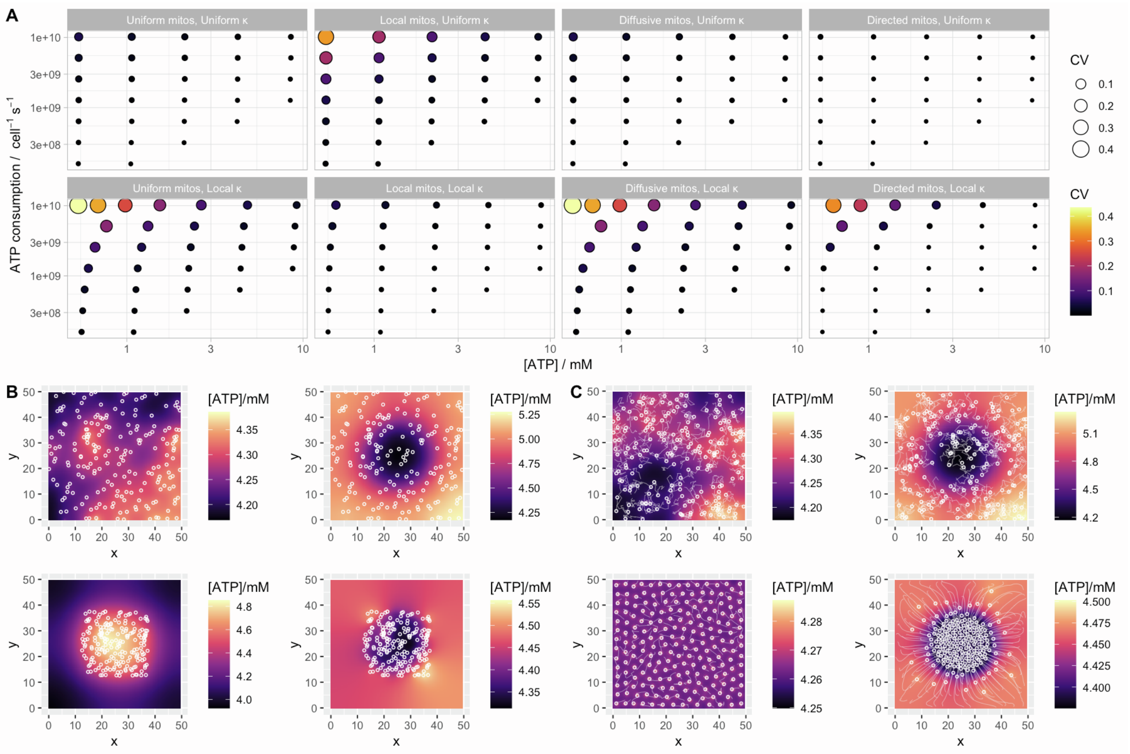

Supplementary Figure 4. **Model with more mitochondria.** 200 mitochondria in the simulated cell. (A) CV of ATP concentration as in Fig. 3. (B) Long-term concentration profile with static mitochondria as in Fig. 2. (C) Long-term concentration profile with static mitochondria as in Fig. 4.

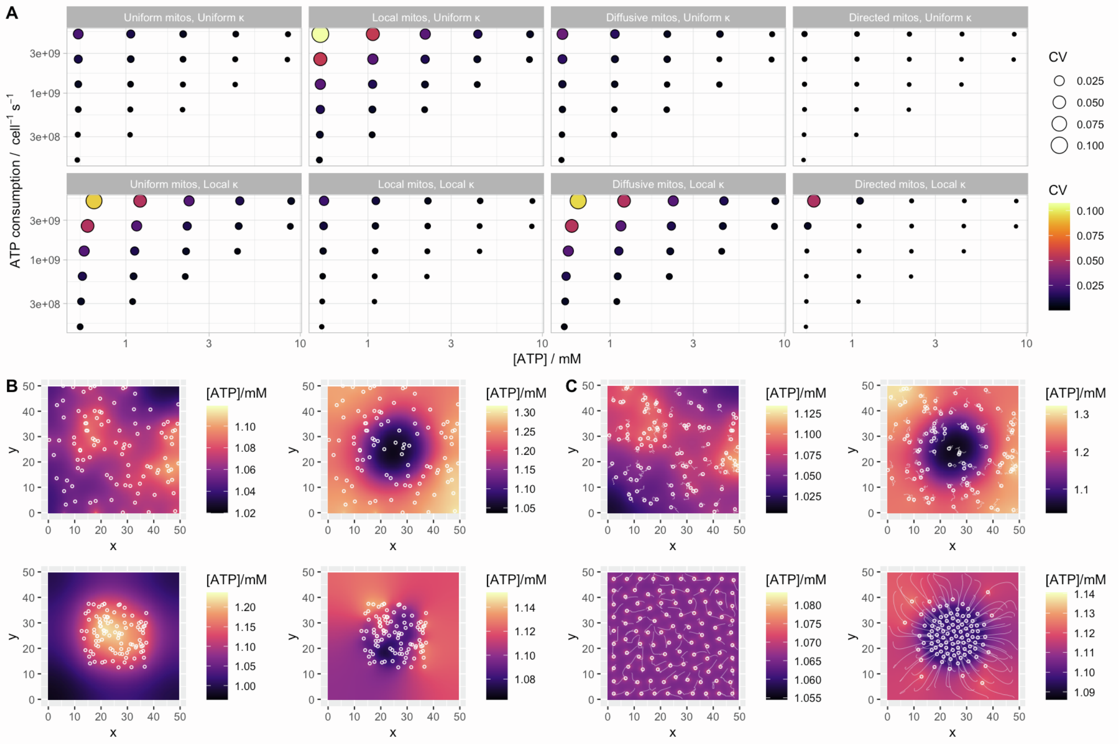

Supplementary Figure 5. **Model with higher cell thickness.** Cell thickness assumed to be 20μm. (A) CV of ATP concentration as in Fig. 3. (B) Long-term concentration profile with static mitochondria as in Fig. 2. (C) Long-term concentration profile with static mitochondria as in Fig. 4.

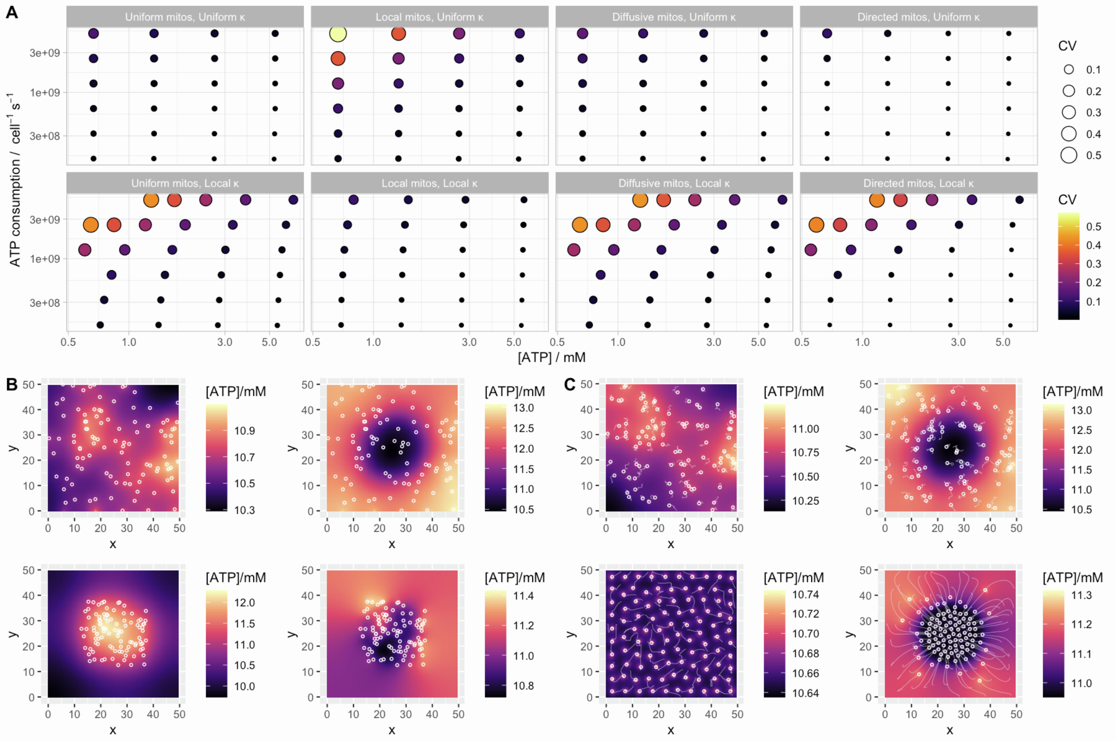

Supplementary Figure 6. **Model with lower cell thickness.** Cell thickness assumed to be 2μm. (A) CV of ATP concentration as in Fig. 3. (B) Long-term concentration profile with static mitochondria as in Fig. 2. (C) Long-term concentration profile with static mitochondria as in Fig. 4.
