# Supplementary material for: Estimating physical conditions supporting gradients of ATP concentration in the eukaryotic cell
Source: Biophys J. 2025 Jun 16;125(2):377–86. doi: 10.1016/j.bpj.2025.06.016 (PMC12968834; doi:10.1016/j.bpj.2025.06.016)
Supplement: Document S2. Article plus Supporting Material [file mmc2.pdf]

Biophysical Journal
Article

Biophysical Society

Estimating physical conditions supporting gradients of ATP concentration in the eukaryotic cell

Rajneesh Kumar[1] and Iain G. Johnston[1,2,*]
[1]Department of Mathematics, University of Bergen, Bergen, Norway and [2]Computational Biology Unit, University of Bergen, Bergen, Norway

ABSTRACT The ATP molecule serves as an energy currency in eukaryotes (and all life), providing the energy needed for many essential cellular processes. But the extent to which substantial spatial differences exist in ATP concentration in the cell remains incompletely known. It is variously argued that ATP diffuses too quickly for large gradients to be established, or that the high rates of ATP production and use (sources and sinks) can support large gradients even with rapid diffusion—and microscopic models and detailed experiments in different specific cases support both pictures. Here, we attempt a mesoscopic investigation, using reaction-diffusion modeling in a simple biophysical picture of the cell to attempt to ask, generally, which conditions cause substantial ATP gradients to emerge within eukaryotic cells. If ATP sources (like mitochondria) or sinks (like the nucleus) are spatially clustered, large fold changes in concentration can exist across the cell; if sources and sinks are more spread, then rapid diffusion indeed prevents large gradients from being established. This dependence holds in model cells of different sizes, suggesting its generality across cell types. Our theoretical work will complement developing intracellular approaches exploring ATP concentration inside eukaryotic cells.

SIGNIFICANCE ATP is a molecule that provides energy for many cellular processes. It is produced and consumed at various sites in eukaryotic cells. The variation of ATP concentration through the cell is an important influence on these processes, but this variation is hard to characterize, and limited experimental information is available. Here, we attempt to provide a general picture of when substantial ATP concentration differences arise in cells, using a simple physical model for reaction and diffusion. This model describes the arrangements and dynamics of ATP sources (like mitochondria) and sinks that lead to uniform or heterogeneous ATP concentration profiles. We connect the model with existing experiments and future directions to further characterize these biophysically important ATP landscapes.

INTRODUCTION

Adenosine triphosphate (ATP) serves as a source of energy for many biochemical reactions throughout the cell. The concentration of ATP (and the ATP:ADP ratio, influencing the free energy available from ATP hydrolysis) determines the energy availability for a given reaction. ATP concentration in animal cells, for example, is often in the range 1–10 mM (1). Cellular ATP concentrations vary through tissues and under different conditions (2). But how much does local ATP concentration vary within a cell? What influences whether substantial spatial gradients of ATP exist across cellular regions—and hence whether energy availability is in a sense heterogeneous throughout the cell?

As a small molecule (consisting of 47 atoms), ATP diffuses rapidly through the cytoplasm (of the order of several hundred $\mu m^2 s^{-1}$, sufficient to cross a 10-μm cell in around 0.2 s). But this does not necessarily mean that the profile of ATP concentration will equilibrate to a spatially uniform level. If localized sources and/or sinks of ATP produce and/or consume ATP at a sufficient rate, arbitrarily large concentration gradients can still exist.

Localized sources of ATP do exist in the eukaryotic cell. Mitochondria are bioenergetic organelles found in the majority of eukaryotic cell types (3) (Fig. 1 *A*). Among many other processes (4), they catalyze the production of ATP from ADP and inorganic phosphate, through the process of oxidative phosphorylation (OXPHOS). OXPHOS is localized to mitochondria and often generates ATP at a greater rate than other cellular processes (including glycolysis), making mitochondria important local sources of ATP (though not under all conditions; see discussion). Mitochondria are highly dynamic within many eukaryotic cells,

Submitted February 20, 2025, and accepted for publication June 13, 2025.
*Correspondence: iain.johnston@uib.no
Editor: Abhishek Singharoy.
https://doi.org/10.1016/j.bpj.2025.06.016
© 2025 The Author(s). Published by Elsevier Inc. on behalf of Biophysical Society.
This is an open access article under the CC BY license (http://creativecommons.org/licenses/by/4.0/).

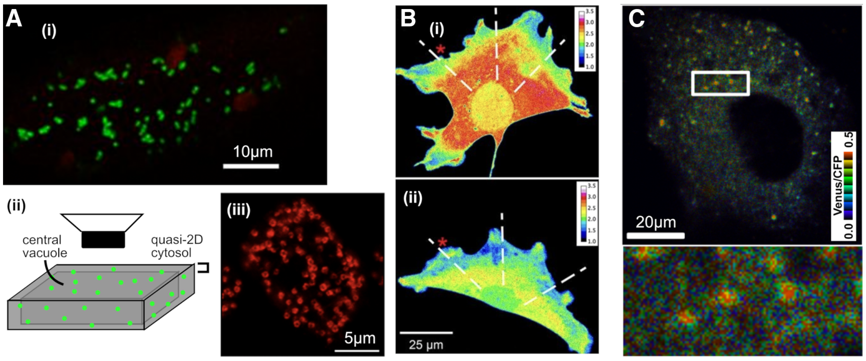

FIGURE 1 Example of cell geometries and ATP-gradient-related experimental results. (*A*) Examples of eukaryotic cell structures with punctate mitochondria. (i) An example plant cell from the hypocotyl of an *Arabidopsis thaliana* (plant) seedling, with discrete, punctate mitochondria visualized using green fluorescent protein (6,43) (*red objects* are chloroplasts). (ii) A schematic structure of plant hypocotyl cells, where a large central vacuole makes the cytosol almost 2D (47); the image from (i) is imaged using a microscope with focal plane aligning with this section. (iii) A *Dictyostelium discoideum* (ameba) cell with mitochondria visualized with antimitoporin from (63). (*B*) A mouse embryonic fibroblast (MEF) cell with ATP:ADP ratio visualized (heatmap) using the PercevalHR probe (64). (i) shows a vertical view as in (A), and (ii) shows a vertical section through the same cell, with smaller dimension and more uniform concentration profile. (*C*) A Huh-7 (immortalized epithelial) cell using the ATeam sensor (33) to report ATP levels (heatmap). Licensing: (A) (iii) is taken from (63) under a CC-BY-NC license and has been edited to add annotations. (B) is taken from (30) under a CC-BY-NC-SA license and has been edited to replace annotations; this subpanel of this figure is therefore also subject to a CC-BY-NC-SA license. (C) is taken from (35) under a CC-BY license and has been edited to move the color scale and add annotation to the length scale bar.

moved by motor proteins on the cytoskeleton, which themselves require ATP. An often-discussed question is the extent to which the speed of mitochondrial motion is directly controlled or a passive result of mitochondrial output providing this ATP supply.

Preserving spacing between mitochondria is hypothesized to be a priority in many cellular cases (5,6), particularly in the context of the cellular society (7,8). Even spacing is valuable for uniform distribution of metabolites and signals through the cell and faithful partitioning of mitochondria at cell divisions (9–14).

ATP concentration features in a large collection of biomathematical models for metabolism and other cellular processes. Such models may use a well-mixed approximation, where concentrations do not vary with spatial coordinates (though may vary with time) and are in a sense averaged across the cell. Some examples include approaches involving ordinary differential equations (15–18), and indeed most “whole-cell models” (19). Other models explicitly represent the concentration of ATP (and associated metabolites) as a function of position within a specific cell type, often with a reaction-diffusion picture (20–23). One recent example is the demonstration that spacing between mitochondria can emerge as a consequence of their influence on and response to the (heterogeneous) ATP profile of the cell in axons (24). Another model, focusing on mitochondrial arrangement in cardiac cells, suggests that ATP concentration variability across the cell is limited in this system—under 10 μM against an average concentration around 10 mM—despite an uneven mitochondrial distribution (20). Similar reaction-diffusion approaches have been used to explore the cellular profiles of other related chemical species including oxygen (25) and calcium (26), and indeed of ATP within the mitochondrion (27). Bridging these targeted approaches and the whole-cell modeling paradigm, spatial algorithms have been recently developed that consider the spatial behavior of ATP (and other metabolites) with specific, detailed cellular structure (28).

Experimental characterization of the within-cell concentration profiles of ATP (and associated metabolites) is typically through fluorescence microscopy (recently reviewed in (29)). Many beautiful approaches have revealed bulk or compartment-specific ATP levels (for example, comparing cytosolic to mitochondrial concentrations); we focus here on those that directly report *spatial variation* in ATP concentration in the cytosol. Groundbreaking work has revealed how mitochondrial position in mouse embryonic fibroblasts directly influences the spatial structure of ATP:ADP ratio within the cell, with the ratio highest in regions of dense mitochondria and with around threefold variation through the cell (30) (Fig. 1 *B*). In agreement, the ATP:ADP ratio in the quasi-1D system of mouse axons decreases with distance from mitochondria (31), which modeling suggests can lead in term to emergent uniform spacing of mitochondria along the axon (24). Semiquantitative visualization of ATP levels in HEK293 (immortalized kidney) cells also shows moderate within-cell ATP differences through the cytosol (32). In HeLa cells and yeast, the scale of heterogeneity in an ATP-related fluorescent signal within cells appears to be of a lower magnitude, though still present (33,34). The same probe was used to identify “hotspots” of ATP colocalized with sites of viral replication, with a fivefold difference in ATP concentration between these hotspots and the cellular background (35) (Fig. 1 *C*).

Our research question here can be thought of as a complement to these results, and an attempt to unite them. The above modeling and experimental work has characterized the existence and magnitude of cytosolic ATP gradients in *specific* cellular circumstances. Here we attempt to explore under what *general* cellular circumstances ATP gradients of a given magnitude could exist. To this end, we require a

system where many different instances can be explored in a computationally reasonable time. We therefore adopt a coarse-grained paradigm, considering a simplified 2D system that we design to retain the important quantitative characteristics determining the magnitude of ATP gradients while also being computationally tractable. For more detailed specific cellular circumstances, more precise modeling approaches may be desirable (28). We adopt the paradigm of "back-of-the envelope biology" (36,37), working with orders-of-magnitude quantities and physical reasoning to gain general numerical estimates of mechanism-linked quantities.

MATERIALS AND METHODS

Equations of motion

Our basic picture is a reaction-diffusion model in a 2D model cell—that is, a model allowing ATP to diffuse and undergo reactions of production and consumption. Our governing partial differential equation is

$$\frac{\partial[\mathrm{ATP}]}{\partial t} = \mathrm{D}\,\nabla^2[ATP] - \kappa(x,y)\,[ATP] + \sum_{i=1}^{n} \rho\,\delta(x - x_i)\delta(y - y_i), \quad (1)$$

encoding the diffusion of ATP through the cell with diffusion constant D, the consumption of ATP with rate $\kappa(x,y)$ throughout the cell, and the production of ATP at rate ρ at a collection of discrete sites (x_i, y_i) corresponding to the n mitochondria in the cell. The consumption term may be applied uniformly throughout the cell or limited to a central region, as described in the text. We consider [ATP] to vary only in 2D but picture a given cell depth to facilitate comparisons of concentration and volume; the third dimension is assumed to be relatively small and to have a uniform ATP profile (Fig. 1 *A* and *B*) (see discussion). By default, we use a simple model cell of 50 μm × 50 μm × 10 μm (hence volume 2.5 × 10^4 μm^3) to model a (relatively large) cell (Fig. 1 *A* and *B*) (5); we also consider 20 μm × 20 μm × 10 μm (hence volume 4 × 10^3 μm^3) to model relatively small eukaryotic cells (38), Bionumber 105879 (39). We also vary cell thickness to assess its contribution to ATP gradient magnitude.

It will immediately be seen that metabolism is effectively ignored in our model; we do not consider the details of the precursors to ATP production or of its consumption and do not consider ADP, the concentration of which determines the free energy associated with the ATP landscape. For simplicity and biophysical generality, we just consider ATP as an independent diffusible metabolite with sources and sinks throughout the cell.

We also neglect ATP production from glycolysis. If oxidative phosphorylation is fully active, the amount of ATP produced from a glucose precursor is over an order of magnitude higher from mitochondria than from glycolysis (40). Additionally, glycolytic ATP production will be more evenly spaced through the cell than mitochondrial ATP production and, hence, contribute less to the establishment of spatial gradients of ATP concentration.

Geometry of ATP sinks

We consider three cases for the spatial dependence of ATP consumption $\kappa(x, y)$. The first case is uniform, $\kappa(x,y) = \kappa$, picturing uniform ATP consumption throughout the cell (for example, where ATP consumption is dominated by translation at evenly spaced ribosomes). The second case has $\kappa(x,y)$ nonzero only in a circular region at the center of the cell, with diameter equal to half the cell width. This arrangement roughly models dominant ATP consumption at the nuclear compartment. The third case has $\kappa(x,y)$ nonzero only along three linear bands across the cell, representing sarcomeres or other linear structures of ATP demand. In each of the latter cases, the magnitude of $\kappa(x,y)$ is scaled by the proportional area involved, so the same overall magnitude of ATP consumption is expected. We allow mitochondria to occupy the same 2D coordinates as these consumption regions to support cases like perinuclear clustering, where mitochondria occupy a shell around the nucleus—which can be pictured in our quasi-2D picture as occupying space "above" or "below" the nuclear region.

Mitochondrial motion

In the case of motile mitochondria, we used two models for motion. Both models describe the physical change in a mitochondrial position vector $\mathbf{x} = (x_i, y_i)$ in one discrete timestep in our numerical simulation. First, random diffusion with diffusion either constant or scaled by ATP:

$$\frac{\Delta \mathbf{x}}{\Delta t} \sim \mathrm{N}\left(0, \Sigma^2\right), \Sigma^2 = \alpha\left(\gamma\,[\mathrm{ATP}] + (1-\gamma)\right) \mathrm{I}, \quad (2)$$

where I is the identity matrix, α is a scaling factor determining the scale of mitochondrial movement, and $\gamma = 0$ for ATP-independent diffusion and $\gamma = 1$ for ATP-dependent diffusion. This picture models mitochondrial motion without systematic directionality, either due to random diffusion ($\gamma = 0$) or random driven motion around the cytoskeleton ($\gamma = 1$). Second, directed motion down the [ATP] gradient:

$$\frac{\Delta \mathbf{x}}{\Delta t} \sim \mathrm{U}(0,\beta)\,\frac{-\nabla[\mathrm{ATP}]}{|\nabla[\mathrm{ATP}]|}, \quad (3)$$

where β is a scaling factor determining the scale of mitochondrial movement, and the uniform random distribution is used both to model fluctuations in dynamics and for numerical convenience. The scaling factors we use are $\alpha = 1.25 \times 10^{-2}$ (for $\gamma = 0$) or 5×10^{-6} (for $\gamma = 1$) and $\beta = 1$, chosen to restrict mitochondrial motion under typical ATP conditions to a maximum speed of order 1 μm s^{-1} (5). We ignore the locally incurred ATP cost of moving mitochondria, which is assumed to be small compared with the other processes consuming ATP in the cell.

Parameterization

We use some parameter values (D) directly from previous observations. Others (κ, ρ) are less directly observable and are constrained through their indirect effect on observable quantities. For these, we assume some bounds on the orders of magnitude involved, and scan through values so that observed quantities take biologically reasonable values.

We assume cellular production of ATP is on the scale of 10^9 ATP/s (41). We assume a default of 100 individual mitochondria in the cell, broadly compatible with observations in plants (5,7,42,43) (Fig. 1 *A*); counts vary dramatically across other species and tissue types, and mitochondria in other clades often fuse into networks (12,44) (see discussion). We vary this value to explore its influence on ATP gradient magnitude. We ignore glycolysis and other sources of ATP production, so that we expect a balancing ATP production per mitochondrion to be on the scale of 10^7 ATP/s/mito. We impose an ATP diffusion rate of 2.5 × 10^2 μm^2 s^{-1} (45); although muscle, the cell type for that observation, likely poses more restrictions to ATP diffusion than other cell types, diffusion in water is the same order of magnitude (7.1× 10^2 μm^2 s^{-1} (46)), so the range for this value appears reasonable.

Typical intracellular ATP concentrations are around 1–10 mM (1). A value of 1 mM corresponds to 10^{-3} mol dm^{-3} = 10^{-3} mol $(10^{15}\ \mu m^3)^{-1}$ = 10^{-3} × 6 × $10^{23}/10^{15}$ = 6 × 10^5 ATP/μm^3. For our example model cell with a depth of 10 μm, this corresponds to a ATP amount per square-micron region of around 6 × 10^4 ATP/μm^2.

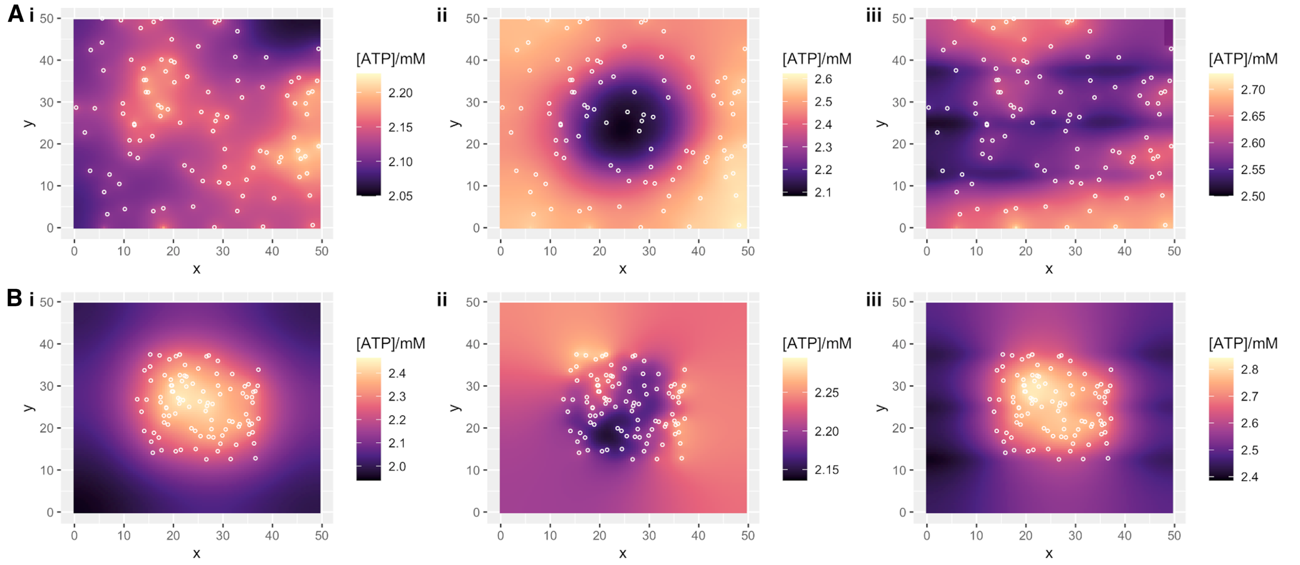

FIGURE 2 Examples of model behavior with static mitochondria exhibiting ATP gradients. Long-term behavior of ATP concentration in the cell under different cellular scenarios with static mitochondria. Plots give x-y dimensions of the model cell in micrometers, with local ATP concentration (*heatmap*) and positions of mitochondria (*circles*). (*A*) Mitochondria arranged uniformly at random in the cell; (*B*) mitochondria arranged uniformly at random in a central cellular region. (i) ATP consumption uniform through the cell ("ribosomes"); (ii) ATP consumption only nonzero in a uniform central circular region ("nucleus"); (iii) ATP consumption only nonzero in uniform horizontal fibers spanning the cell ("sarcomeres"). Parameterizations were chosen to give comparable ATP concentrations around 2 mM in each case; they correspond to cellular consumption rates around 5.1×10^9 molecules s^{-1}.

Numerics

We use a simple finite difference solver with a timestep of 2.5×10^{-4} s and grid element size of 0.5 μm, initializing the cell with zero ATP concentration throughout, and we simulate the evolution of the system until a steady-state criterion is met (no relative ATP change greater than 10^{-4} across the domain). For comparison with biological criteria, we report the total consumption rate of ATP throughout the cell and the total ATP amount in the cell.

As mentioned above, we work in a quasi-2D picture, where the depth of the cytoplasm is less important than the length and width. This is not unreasonable in, for example, plant hypocotyl cells, where the cytoplasm can be almost arbitrarily thin (Fig. 1 *A*) (5,47). It can also be pictured as an approximation to cells that are relatively flat compared with their diameter, and where smaller ATP variability exists in the direction of depth than in other directions (Fig. 1 *B*). In this situation, we neglect ATP variation in the depth direction and consider the 2D case where the concentration reflects an integrated value across cell depth.

Code

All code is freely available at https://github.com/StochasticBiology/mito-agents. We use custom C code to simulate the model and R (48) with libraries *dplyr* (49), *ggplot2* (50), *ggpubr* (51), and *viridis* (52) for visualization.

RESULTS

Influence of model source-sink distributions with static mitochondria

We first scanned through parameters (κ, ρ) (ATP consumption and production) to identify parameterizations compatible with biological values of cellular ATP content and consumption. We focus on parameterizations that, under equilibrium conditions, give cellular consumption values between 10^8 ATP/s and 10^{10} ATP/s, and ATP concentration around 0.5–10 mM (see materials and methods and (1,41)).

We consider several different arrangements of ATP sources (mitochondria) and sinks. We either randomly space mitochondria uniformly throughout the cell or cluster them in the center; and we either space ATP consumption uniformly throughout the cell (modeling, for example, evenly spaced ribosomes), restrict it to a central circular region (modeling, for example, the nucleus), or restrict in to linear bands spanning the cell (modeling, for example, sarcomeres) (see materials and methods; Fig. 2). In all cases, we run the simulated cell until ATP concentration reaches a steady state, and then we report the coefficient of variation (CV)—the ratio of the standard deviation of ATP concentration to the mean across in the cell (Fig. 3). Equilibration times were generally rapid under all parameterizations we considered. From an unphysical initial condition of zero ATP concentration, simulations typically reached a steady state ATP profile in under 30 s of simulation time.

We found that strong determinants of ATP gradient magnitude were the overall concentration of ATP (lower concentrations supporting higher gradients) and the cellular rate of ATP consumption (higher consumptions supporting higher gradients). Substantial gradients with CVs over 10% typically occur only at the limits of our biological window for these values (Fig. 3). At given amounts of concentration and consumption, we found (following intuition) that the magnitude of ATP fold change is dependent strongly on

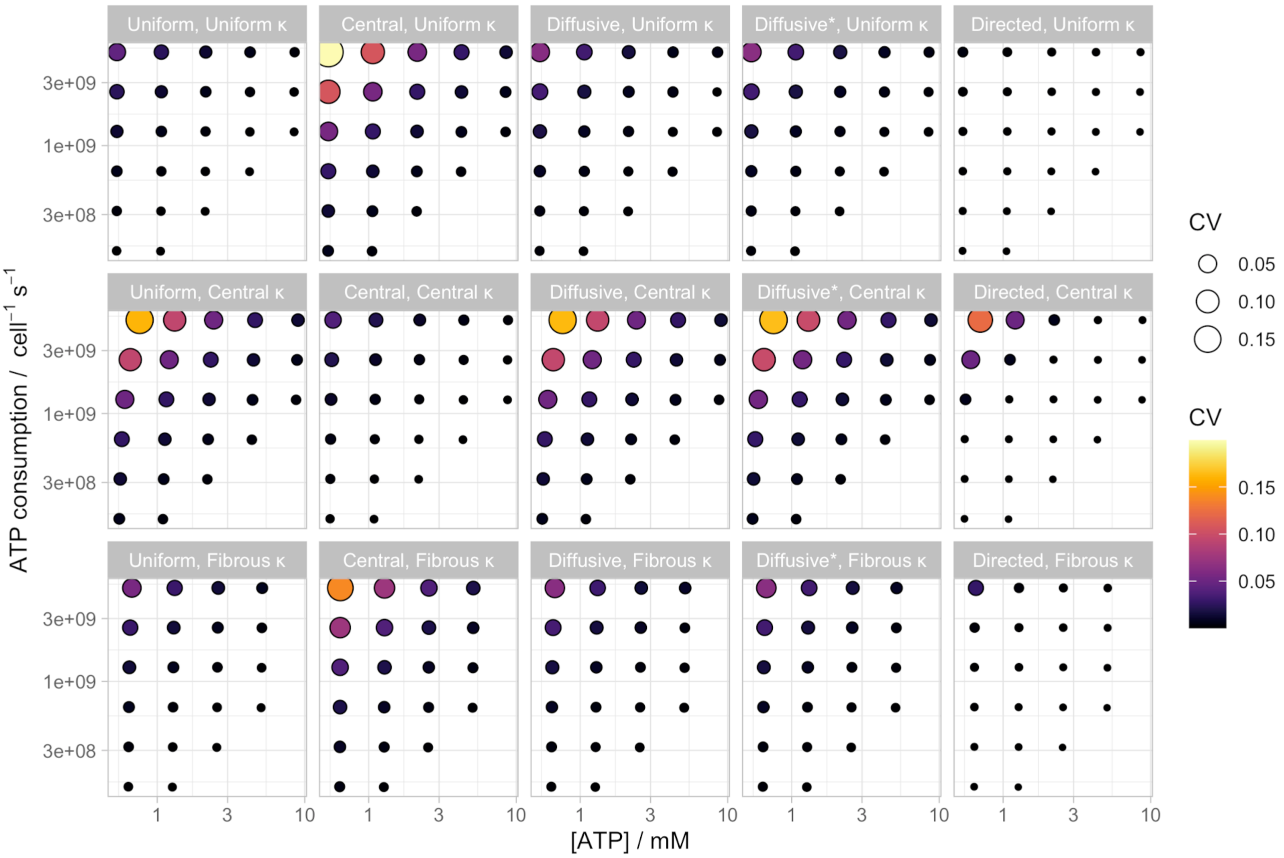

FIGURE 3 Model ATP concentration differences under different conditions. Coefficient of variation (CV) of ATP concentration across the cell with cellular ATP concentration and ATP consumption rate. Labels describe mitochondria uniformly randomly distributed and static (uniform), clustered locally at the center of the cell (local), undergoing diffusive motion (diffusive for ATP-independent, diffusive* for ATP-dependent motion, Eq. 2), and undergoing directed motion (directed, Eq. 3); and ATP consumption (κ) uniform through the cell (uniform), localized in the center of the cell (central), or in horizontal fibers (fibrous). These CV ranges correspond roughly to fold-change values between 0 and 2.5 (Fig. S1).

the arrangement of cellular sources and sinks. If static mitochondria and ATP consumption are "matched"—either both evenly spaced or colocalized in the central region of the cell—no combination of parameters compatible with biological observations led to more than a 10% CV in ATP across the cell, and most led to substantially smaller gradients (Figs. 2 *A*i, *B*ii, and 3). However, if the positions of mitochondria and ATP consumption were less closely correlated, CV in ATP concentration across the cell could reach rather higher levels >15% (Figs. 2 *A*ii, *B*i, and 3). The CV was highly correlated with the fold range between the minimum and the maximum ATP concentrations found across the cell: these higher CV cases correspond to an over twofold difference between minimum and maximum ATP concentration (Figs. 2 and S1). These results did not change substantially when we considered smaller cell sizes (Fig. S2). The behavior of the "fibrous" ATP consumption case (Fig. 2 iii), qualitatively reflecting an intermediate between uniformly spaced and centrally localized ATP consumption, correspondingly showed intermediate CV behaviors between these two cases, albeit more resembling the uniform consumption case.

Model behavior with dynamic mitochondria

We next allowed mitochondria to move in the cell, following one of two models: random diffusion, potentially scaled by local ATP concentration (Eq. 2) or directed motion down the local ATP gradient (Eq. 3). Example dynamics and ATP profiles are shown in Fig. 4. No dynamic protocol qualitatively changed our results on ATP gradients (Fig. 3), although several emergent dynamics were clear. When mitochondria were actively moved following an ATP gradient, even spacing throughout the cell emerged in the case of uniform ATP consumption (Fig. 4 *C*), following the observation of (24) that the coupled production-motion system naturally leads to mitochondrial spacing (Fig. 4 *C*i). In the case of localized ATP consumption, mitochondria in the central model moved preferentially toward the center of the cell to set up "perinuclear" clustering with even spacing (Fig. 4 *C*ii) and toward fibers in the "fibrous" model (Fig. 4 *C*iii). Again, results were consistent in the case of smaller cells (Fig. S2).

One interesting specific comparison is between the artificially placed, centrally clustered static mitochondria (Fig. 2 *B*ii) and the directed-motion mitochondria (Fig. 4 *C*ii).

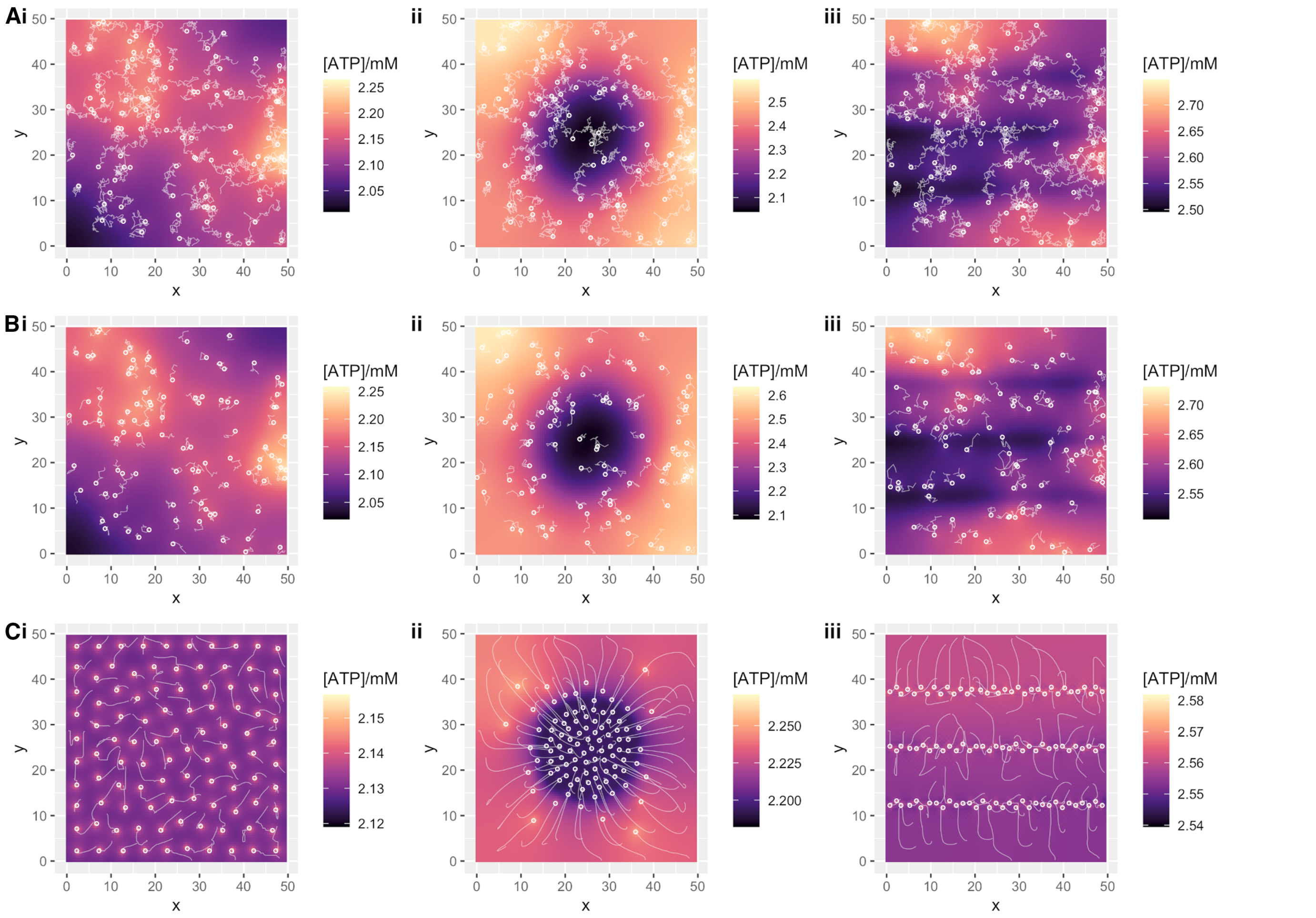

FIGURE 4 Model ATP behavior with mitochondrial dynamics. Example long-term behavior of ATP concentration in the cell under different cellular scenarios with dynamic mitochondria. Plots give x-y dimensions of the model cell in micrometers, with local ATP concentration (*heatmap*) and positions of mitochondria (*circles*). Light trails show mitochondrial motion. (*A*) Mitochondria diffusing independent of ATP; (*B*) mitochondria diffusing with ATP-dependent diffusion rate; (*C*) mitochondria undergoing directed motion down the ATP gradient. (i) Uniform ATP consumption; (ii) ATP consumption in central circular region; (iii) ATP consumption in horizontal fibers. For (*A*)–(*B*), trails show mitochondria motion over the final 10 s of simulation; for (*C*), trials show mitochondrial motion throughout the simulation (from random initial conditions to a stable arrangement). Parameterizations were chosen to give comparable ATP concentrations around 2 mM in each case; they correspond to cellular consumption rates around 5.1×10^9 molecules s^{-1}.

Fig. 3 shows that the CV for the directed mitochondria is marginally higher than in the static case. Here, although most directed mitochondria end up evenly spaced in the center of the cell, some appear to become immobilized away from the center, presumably because the ATP produced by more central mitochondria "mask" the cell-scale gradient from the local of these peripheral mitochondria. These immobilized individuals then make the population less completely aligned with the ATP-consuming region than in the artificially arranged case, and the supply and demand are less perfectly matched, increasing CV.

Influence of model cell dimensions and mitochondrial number

We next asked how cell thickness and mitochondrial number influenced the behavior of ATP gradients in the cell. To this end, we simulated (only using the uniform and central ATP consumption models) behavior with fewer (50, Fig. S3) and more (200, Fig. S4) mitochondria, and with thinner (2 μm, Fig. S5) and thicker (20 μm, Fig. S6) dimensions in the "depth" direction. In each case the qualitative direction of the results is the same; lower ATP concentrations and higher consumption rates give higher CVs for ATP, and localized energy demand without compensatory mitochondrial localization gives the highest CV magnitudes.

The number of mitochondria in our model did not lead to a notable change in the scale of model CVs at a given concentration-consumption state (Figs. 3, S3 *A*, and S4 *A*). For example, the CV for diffusive mitochondria and centralized demand at an ATP concentration of 1 mM and a consumption rate of 10^9 molecules s^{-1} is typically around 0.05 for all the mitochondrial numbers we consider; the most extreme case shared by all experiments, with clustered

mitochondria, uniform demand, [ATP] around 0.5 mM, and consumption 3×10^9 s^{-1}, had a CV around 0.12 in all cases. The thickness of the cell in our model had a stronger effect. At 1 mM concentration and 10^9 molecules s^{-1} consumption, the thinner cell had a CV around 0.25 and the thicker cell around 0.02, suggesting an inverse linear scaling of CV with cell thickness.

We verified that for a given (κ, ρ) parameterization of the model, the cell depth did not influence the number of ATP molecules per simulation element (as the cell depth does not feature in the simulation itself, only playing a role in the post hoc calculation of concentration from this quantity). However, a given number of ATP molecules per model area element will translate to a higher concentration in a thinner cell and a lower concentration in a thicker cell. The (κ, ρ) parameterization corresponding to a given cellular ATP concentration will thus depend on cell thickness: broadly, thinner cells achieve a given ATP concentration with lower production or higher consumption terms. The increase in CV at lower model thicknesses for a given cellular ATP concentration can then be explained by the change in the rates of the production and consumption processes needed to achieve that concentration for a given cell thickness.

DISCUSSION

In this simple biophysical model, after the concentration and consumption of ATP, the relative positioning of ATP sources and sinks strongly influences whether substantial ATP concentration gradients exist in the cell. Under reasonable biological parameterizations, it is possible either for negligible differences (CV $<$ 1%) to exist across the cell or to have fairly substantial concentration differences (CV $>$ 15%, twofold changes across the cell). A notable example of this structure-induced difference is in the low-concentration, high-consumption region of Fig. 3; with static, clustered mitochondria and uniform consumption, CV exceeds 15%, whereas with dynamic mitochondria that respond to the ATP gradient, CV is almost negligible (resembling Fig. 4 *C*). The specific scales of CV depend on the thickness of the model cell, with thinner cytoplasmic sections supporting proportionally higher CV values and vice versa. The dependence of CV on mitochondrial number (and density) is much more limited. Across all models, CVs are highest when ATP concentrations are lowest and consumption rates are highest. Agreeing with experimental observations (30,31) and intuition, the highest ATP concentrations are found in regions of densely arranged mitochondria. We believe that the insights from this model can help explain the diversity of experimental observations described in the introduction: under different cellular arrangements and mitochondrial arrangements and dynamics, cellular ATP gradients may be relatively limited or rather pronounced. In the largest cases, the severalfold change predicted by our model is in reasonable correspondence with the severalfold changes observed in experiments detecting ATP heterogeneity (33,34). We do not observe as extreme results as the fivefold differences in (35), but the cellular circumstances underlying those observations (localized replication of viral genomes) have no equivalent in our model and may require more dedicated and detailed modeling to capture.

Heterogeneity in ATP profile (where it existed) had a substantial effect on the motion of mitochondria. In the case of directed motion down the ATP gradient, mitochondria rapidly adopted positioning that reflected the spread or localization of demand (Fig. 4 *C*). We saw little evidence under passive diffusion that differences in ATP concentration across the cell led to pronounced emergence of mitochondrial structure; for example, there was little evidence that slower mitochondrial motion in lower-concentration regions of the cell led to a “buildup” of mitochondria in those regions (Fig. 4 *B*). Active transport of mitochondria down the concentration gradient was required for such emergent structure. In this situation, we particularly saw the emergence of even spacing regardless of the geometry of the region of demand—matching the “active thermodynamic force” driving even spacing from (24). This spatial heterogeneity in macroscopic cellular components, emerging from variability in an underlying concentration field, underlines the potential importance of considering spatial profiles in “whole-cell modeling” pictures (19). Work considering the optimal arrangement of enzymes using principles of optimization (and operations research) suggests a promising framework for such consideration (7,53,54).

Our model is obviously a very coarse-grained representation of a cell. ATP metabolism is represented simply by point production and proportional degradation, with no representation of ADP, mitochondrial heterogeneity (44), or other degrees of freedom. The geometry of the cell is fixed, and the ATP heterogeneity in the third dimension is effectively ignored. This final point is for computational simplicity, avoiding fully 3D simulations. In several eukaryotic cases, a thin cytoplasm assumption is quite reasonable: for example, the plant hypocotyl cells in Fig. 1, where a central vacuole forces the cytoplasm into a thin layer against the cell wall (5,47). Other cell types, from human lines to protists, also have relatively flat profiles, in culture and in vivo—but this is certainly a simplification that does not generalize across all cells. However, we believe this simplified picture both captures the most essential drivers of ATP concentration gradients and retains interpretability without including the large number of additional parameters that would be required to capture these effects. This simplified philosophy is also clear in our treatment of model parameters. Across eukaryotes, cell size, mitochondrial number, ATP production and consumption, and cell geometries will of course vary dramatically. Some of these variables are, by their nature, difficult to directly observe. Our approach has been one of “back-of-the-envelope” biology: establish an order-of-magnitude estimate (usually from one

or more model organisms) and then scan through possible values that represent at least an order-of-magnitude either side of this, in order to include a wide range of plausible values and capture the general trends in behavior with the parameters of interest. However, there will always be cases where such estimates and ranges do not well capture a specific instance. Modeling approaches at a finer-grained microscopic scale are being developed (28) and provide an alternative to our general mesoscopic picture for specific cellular circumstances.

In different specific situations, mitochondria will not be the dominant source of ATP in the cell. Green plant tissues in the light, for example, generate substantial ATP from chloroplasts (also localized organelles, though often tightly packed throughout the body of such cells). Connecting spatial models of ATP heterogeneity to established, more detailed models of ATP behavior in photosynthetic cells (55,56) (and, indeed, other cell types (15)) is an interesting future modeling target. In low-oxygen conditions or nutrient substrates that do not support OXPHOS, other processes like glycolysis may be the more important sources of ATP, reflecting another future axis of investigation.

The fragmented nature of our model mitochondria is, of course, not representative of all cell types in all organisms. Although mitochondria exist in a predominantly fragmented form in, for example, somatic plant cells (42,43), in other organisms they fuse into larger connected networks (12). If mitochondria instead form a reticulated network along which ATP production is relatively uniform, ATP sources will be more evenly spread through the cell, and concentration gradients will be diminished further. Spatially extended mitochondria, where ATP production occurs more uniformly along elongated tubules/networks, will intuitively lead to more uniform ATP concentration profiles compared with the punctate case. As capturing network structure would involve a collection of additional degrees of freedom (for example, fused proportion, branching rate, and so on (12,57–61)), we reserve this for future work: generalization of this simple model to support more connected mitochondrial elements and/or elements beyond simple point sources could readily address this connected picture in future.

Looking to future experimental work, given the rate of development of beautiful and powerful approaches for subcellular bioenergetic characterization (29,62), it is conceivable that local ATP concentration measurements will become easier and higher throughput in the near future. The core prediction of our model—how the scale of ATP heterogeneity depends on the relative geometry of mitochondria and ATP sinks—could then be readily tested experimentally. This could be achieved, for example, by jointly recording mitochondrial structure and ATP profiles in cells with a diversity of mitochondrial arrangements (particularly perinuclear clustering vs. even spacing). The computational tractability of our model would also allow it to be tailored to the particular circumstances of a given experiment for more detailed, targeted predictions and insights.

ACKNOWLEDGMENTS

This project has received funding from the European Research Council (ERC) under the European Union's Horizon 2020 research and innovation program (grant agreement no. 805046 (EvoConBiO) to I.G.J.).

AUTHOR CONTRIBUTIONS

I.G.J. designed research. R.K. and I.G.J. performed research and analyzed data. R.K. and I.G.J. wrote the manuscript.

DECLARATION OF INTERESTS

The author declares no competing interests.

SUPPORTING MATERIAL

Supporting Material can be found online at https://doi.org/10.1016/j.bpj.2025.06.016.

REFERENCES

1. Greiner, J. V., and T. Glonek. 2021. Intracellular ATP Concentration and Implication for Cellular Evolution. *Biology.* 10:1166.
2. De Col, V., P. Fuchs, …, M. Schwarzländer. 2017. ATP sensing in living plant cells reveals tissue gradients and stress dynamics of energy physiology. *eLife.* 6:e26770.
3. Roger, A. J., S. A. Muñoz-Gómez, and R. Kamikawa. 2017. The Origin and Diversification of Mitochondria. *Curr. Biol.* 27:R1177–R1192.
4. Picard, M., and O. S. Shirihai. 2022. Mitochondrial signal transduction. *Cell Metab.* 34:1620–1653.
5. Chustecki, J. M., D. J. Gibbs, …, I. G. Johnston. 2021. Network analysis of Arabidopsis mitochondrial dynamics reveals a resolved tradeoff between physical distribution and social connectivity. *Cell Syst.* 12:419–431.e4.
6. Chustecki, J. M., R. D. Etherington, …, I. G. Johnston. 2022. Altered collective mitochondrial dynamics in the Arabidopsis msh1 mutant compromising organelle DNA maintenance. *J. Exp. Bot.* 73:5428–5439.
7. Chustecki, J. M., and I. G. Johnston. 2024. Collective mitochondrial dynamics resolve conflicting cellular tensions: From plants to general principles. *Semin. Cell Dev. Biol.* 156:253–265.
8. Wang, S., and S. Mukherji. 2022. Uncovering the principles coordinating systems-level organelle biogenesis with cellular growth. Preprint at bioRxiv. https://doi.org/10.1101/2022.11.01.514705.
9. Aryaman, J., C. Bowles, …, I. G. Johnston. 2019. Mitochondrial Network State Scales mtDNA Genetic Dynamics. *Genetics.* 212:1429–1443.
10. Edwards, D. M., E. C. Røyrvik, …, I. G. Johnston. 2021. Avoiding organelle mutational meltdown across eukaryotes with or without a germline bottleneck. *PLoS Biol.* 19:e3001153.
11. Glastad, R. C., and I. G. Johnston. 2023. Mitochondrial network structure controls cell-to-cell mtDNA variability generated by cell divisions. *PLoS Comput. Biol.* 19:e1010953.
12. Hoitzing, H., I. G. Johnston, and N. S. Jones. 2015. What is the function of mitochondrial networks? A theoretical assessment of hypotheses and proposal for future research. *Bioessays.* 37:687–700.

13. Jajoo, R., Y. Jung, …, J. Paulsson. 2016. Accurate concentration control of mitochondria and nucleoids. *Science.* 351:169–172.
14. Moore, A. S., S. M. Coscia, …, E. L. F. Holzbaur. 2021. Actin cables and comet tails organize mitochondrial networks in mitosis. *Nature.* 591:659–664.
15. Beard, D. A., and H. Qian. 2008. Chemical Biophysics: Quantitative Analysis of Cellular Systems. Cambridge University Press, Cambridge.
16. Forrest, J., M. Pan, …, M. P. Stumpf. 2023. Energy Dependence of Signalling Dynamics and Robustness in Bacterial Two Component Systems. Preprint at bioRxiv. https://doi.org/10.1101/2023.02.12.528212.
17. Kerr, R., S. Jabbari, and I. G. Johnston. 2019. Intracellular Energy Variability Modulates Cellular Decision-Making Capacity. *Sci. Rep.* 9:20196.
18. Kumar, R., and I. G. Johnston. 2024. ATP dependence of decision-making capacity in a fine-grained model of gene regulatory networks. *J. R. Soc. Interface.* 21:20240246.
19. Goldberg, A. P., B. Szigeti, …, J. R. Karr. 2018. Emerging whole-cell modeling principles and methods. *Curr. Opin. Biotechnol.* 51:97–102.
20. Ghosh, S., K. Tran, …, V. Rajagopal. 2018. Insights on the impact of mitochondrial organisation on bioenergetics in high-resolution computational models of cardiac cell architecture. *PLoS Comput. Biol.* 14:e1006640.
21. Hatano, A., J. i. Okada, …, S. Sugiura. 2011. A Three-Dimensional Simulation Model of Cardiomyocyte Integrating Excitation-Contraction Coupling and Metabolism. *Biophys. J.* 101:2601–2610.
22. Hatano, A., J. I. Okada, …, S. Sugiura. 2015. Distinct Functional Roles of Cardiac Mitochondrial Subpopulations Revealed by a 3D Simulation Model. *Biophys. J.* 108:2732–2739.
23. Vendelin, M., O. Kongas, and V. Saks. 2000. Regulation of mitochondrial respiration in heart cells analyzed by reaction-diffusion model of energy transfer. *Am. J. Physiol. Cell Physiol.* 278:C747–C764.
24. Kajita, M. K., Y. Konishi, and T. S. Hatakeyama. 2024. Active thermodynamic force driven mitochondrial alignment. *Phys. Rev. Res.* 6:L022024.
25. Sedlack, A. J. H., R. Penjweini, …, J. R. Knutson. 2022. Computational Modeling and Imaging of the Intracellular Oxygen Gradient. *Int. J. Mol. Sci.* 23:12597.
26. Colman, M. A., E. Alvarez-Lacalle, …, J. Heijman. 2022. Multi-Scale Computational Modeling of Spatial Calcium Handling From Nanodomain to Whole-Heart: Overview and Perspectives. *Front. Physiol.* 13:836622.
27. Garcia, G. C., T. M. Bartol, …, A. Skupin. 2019. Mitochondrial morphology provides a mechanism for energy buffering at synapses. *Sci. Rep.* 9:18306.
28. Francis, E. A., J. G. Laughlin, …, P. Rangamani. 2024. Spatial modeling algorithms for reactions and transport (SMART) in biological cells. *Nat. Comput. Sci.* 5:76–89.
29. San Martín, A., R. Arce-Molina, …, P. Y. Sandoval. 2022. Visualizing physiological parameters in cells and tissues using genetically encoded indicators for metabolites. *Free Radic. Biol. Med.* 182:34–58.
30. Schuler, M. H., A. Lewandowska, …, B. Cunniff. 2017. Miro1-mediated mitochondrial positioning shapes intracellular energy gradients required for cell migration. *Mol. Biol. Cell.* 28:2159–2169.
31. Matsumoto, N., I. Hori, …, Y. Konishi. 2022. Intermitochondrial signaling regulates the uniform distribution of stationary mitochondria in axons. *Mol. Cell. Neurosci.* 119:103704.
32. White, D., L. Lauterboeck, …, Q. Yang. 2023. Real-Time Visualization of Cytosolic and Mitochondrial ATP Dynamics in Response to Metabolic Stress in Cultured Cells. *Cells.* 12:695.
33. Imamura, H., K. P. H. Nhat, …, H. Noji. 2009. Visualization of ATP levels inside single living cells with fluorescence resonance energy transfer-based genetically encoded indicators. *Proc. Natl. Acad. Sci. USA.* 106:15651–15656.
34. Takaine, M., M. Ueno, …, S. Yoshida. 2019. Reliable imaging of ATP in living budding and fission yeast. *J. Cell Sci.* 132:jcs230649.
35. Ando, T., H. Imamura, …, T. Suzuki. 2012. Visualization and Measurement of ATP Levels in Living Cells Replicating Hepatitis C Virus Genome RNA. *PLoS Pathog.* 8:e1002561.
36. Johnston, I. G., B. C. Rickett, and N. S. Jones. 2014. Explicit Tracking of Uncertainty Increases the Power of Quantitative Rule-of-Thumb Reasoning in Cell Biology. *Biophys. J.* 107:2612–2617.
37. Phillips, R., and R. Milo. 2009. A feeling for the numbers in biology. *Proc. Natl. Acad. Sci. USA.* 106:21465–21471.
38. Puck, T. T., P. I. Marcus, and S. J. Cieciura. 1956. CLONAL GROWTH OF MAMMALIAN CELLS IN VITRO : GROWTH CHARACTERISTICS OF COLONIES FROM SINGLE HELA CELLS WITH AND WITHOUT A "FEEDER" LAYER. *J. Exp. Med.* 103:273–283.
39. Milo, R., P. Jorgensen, …, M. Springer. 2010. BioNumbers—the database of key numbers in molecular and cell biology. *Nucleic Acids Res.* 38:D750–D753.
40. Mookerjee, S. A., A. A. Gerencser, …, M. D. Brand. 2017. Quantifying intracellular rates of glycolytic and oxidative ATP production and consumption using extracellular flux measurements. *J. Biol. Chem.* 292:7189–7207.
41. Flamholz, A., R. Phillips, and R. Milo. 2014. The quantified cell. *Mol. Biol. Cell.* 25:3497–3500.
42. Logan, D. C. 2010. Mitochondrial fusion, division and positioning in plants. *Biochem. Soc. Trans.* 38:789–795.
43. Logan, D. C., and C. J. Leaver. 2000. Mitochondria-targeted GFP highlights the heterogeneity of mitochondrial shape, size and movement within living plant cells. *J. Exp. Bot.* 51:865–871.
44. Aryaman, J., I. G. Johnston, and N. S. Jones. 2019. Mitochondrial Heterogeneity. *Front. Genet.* 9:718.
45. Hubley, M. J., R. C. Rosanske, and T. S. Moerland. 1995. Diffusion coefficients of ATP and creatine phosphate in isolated muscle: pulsed gradient 31P NMR of small biological samples. *NMR Biomed.* 8:72–78.
46. Bowen, W. J., and H. L. Martin. 1964. The diffusion of adenosine triphosphate through aqueous solutions. *Arch. Biochem. Biophys.* 107:30–36.
47. Lee Erickson, J., J. Prautsch, …, M. Schattat. 2023. Stromule geometry allows optimal spatial regulation of organelle interactions in the quasi-2D cytoplasm. Preprint at bioRxiv. https://doi.org/10.1101/2023.04.27.538610.
48. R Core Team. 2022. R: A Language and Environment for Statistical Computing. R Foundation for Statistical Computing, Vienna, Austria.
49. Wickham, H., R. François, …, D. Vaughan. 2023. Dplyr: A Grammar of Data Manipulation. R Package Version 1.1. 2. Computer software.
50. Wickham, H. 2016. ggplot2: Elegant Graphics for Data Analysis. Springer-Verlag.
51. Kassambara, A. 2020. ggpubr:"ggplot2" Based Publication Ready Plots. R Package Version 0.4. 0. 438.
52. Garnier, S., N. Ross, … C. Scherer. 2021. Viridis-Colorblind-Friendly Color Maps for R. R Package Version 0.6. 2.
53. Giunta, G., F. Tostevin, …, U. Gerland. 2022. Optimal spatial allocation of enzymes as an investment problem. *Commun. Phys.* 5:1–12.
54. Sutherland, W. J. 2005. The best solution. *Nature.* 435:569.
55. Vershubskii, A. V., V. I. Mishanin, and A. N. Tikhonov. 2014. Modeling of the photosynthetic electron transport regulation in cyanobacteria. *Biochem. Moscow. Suppl. Ser. A.* 8:262–278.
56. Vershubskii, A. V., V. I. Priklonskii, and A. N. Tikhonov. 2006. Kinetic model for electron and proton transport in chloroplasts with a nonuniform distribution of protein complexes in thylakoid membranes. *Russ. J. Phys. Chem.* 80:467–474.
57. Chuphal, P., J. D. Lanctôt, …, A. I. Brown. 2024. Mitochondrial Network Branching Enables Rapid Protein Spread with Slower Mitochondrial Dynamics. *PRX Life.* 2:043005.
58. Holt, K. B., J. Winter, …, E. F. Koslover. 2024. Spatiotemporal Modeling of Mitochondrial Network Architecture. *PRX Life.* 2:043002.

59. Sukhorukov, V. M., D. Dikov, …, M. Meyer-Hermann. 2012. Emergence of the Mitochondrial Reticulum from Fission and Fusion Dynamics. *PLoS Comput. Biol.* 8:e1002745.

60. Viana, M. P., A. I. Brown, …, S. M. Rafelski. 2020. Mitochondrial Fission and Fusion Dynamics Generate Efficient, Robust, and Evenly Distributed Network Topologies in Budding Yeast Cells. *Cell Syst.* 10:287–297.e5.

61. Zamponi, N., E. Zamponi, …, D. R. Chialvo. 2018. Mitochondrial network complexity emerges from fission/fusion dynamics. *Sci. Rep.* 8:363.

62. Scherschel, M., J.-O. Niemeier, …, B. Morgan. 2024. A family of NADPH/NADP+ biosensors reveals in vivo dynamics of central redox metabolism across eukaryotes. *Nat. Commun.* 15:10704.

63. Rai, A., H. Nöthe, …, D. J. Manstein. 2011. Dictyostelium dynamin B modulates cytoskeletal structures and membranous organelles. *Cell. Mol. Life Sci.* 68:2751–2767.

64. Tantama, M., J. R. Martínez-François, …, G. Yellen. 2013. Imaging energy status in live cells with a fluorescent biosensor of the intracellular ATP-to-ADP ratio. *Nat. Commun.* 4:2550.

Biophysical Journal, Volume *125*

Supplemental information

Estimating physical conditions supporting gradients of ATP concentration in the eukaryotic cell

Rajneesh Kumar and Iain G. Johnston

Supplementary Information

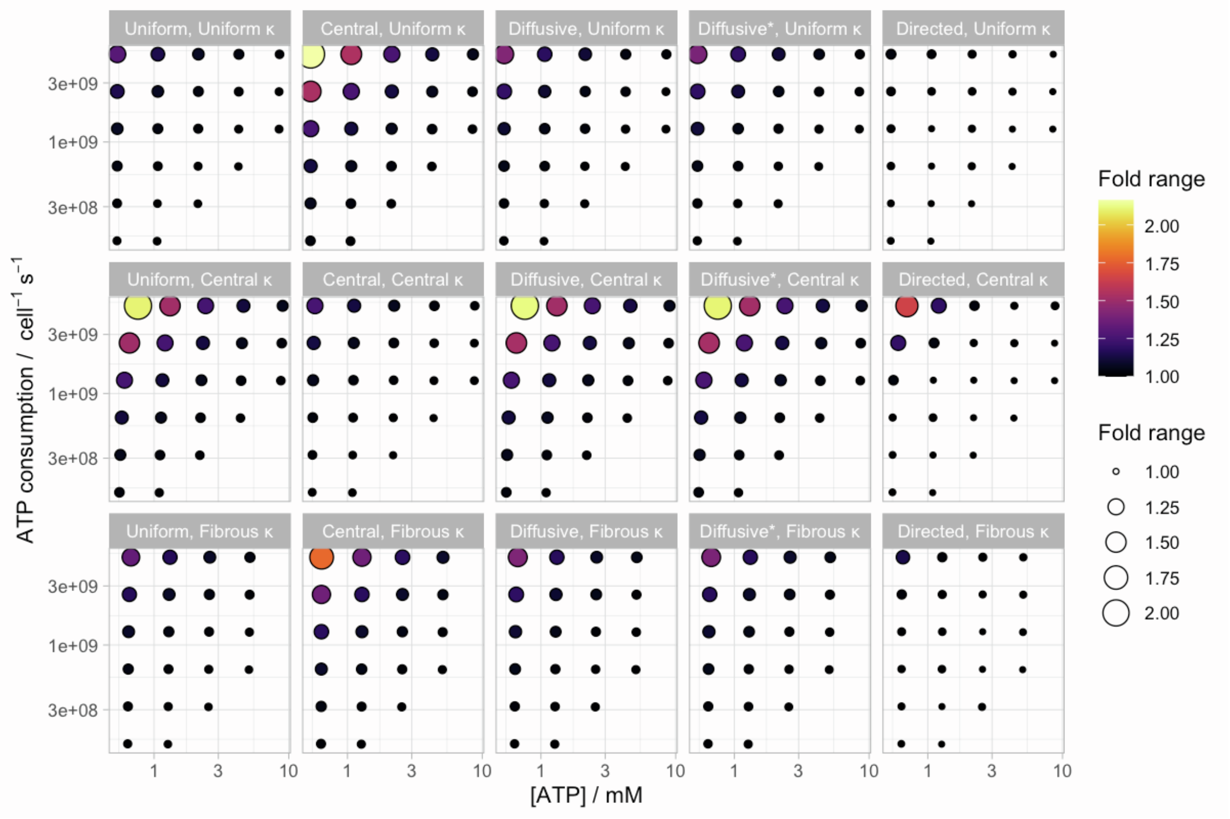

Supplementary Figure 1. **Fold-change in ATP concentration.** ATP concentration gradients plotted as fold change between minimum and maximum values in the cell, rather than CV as in the main text.

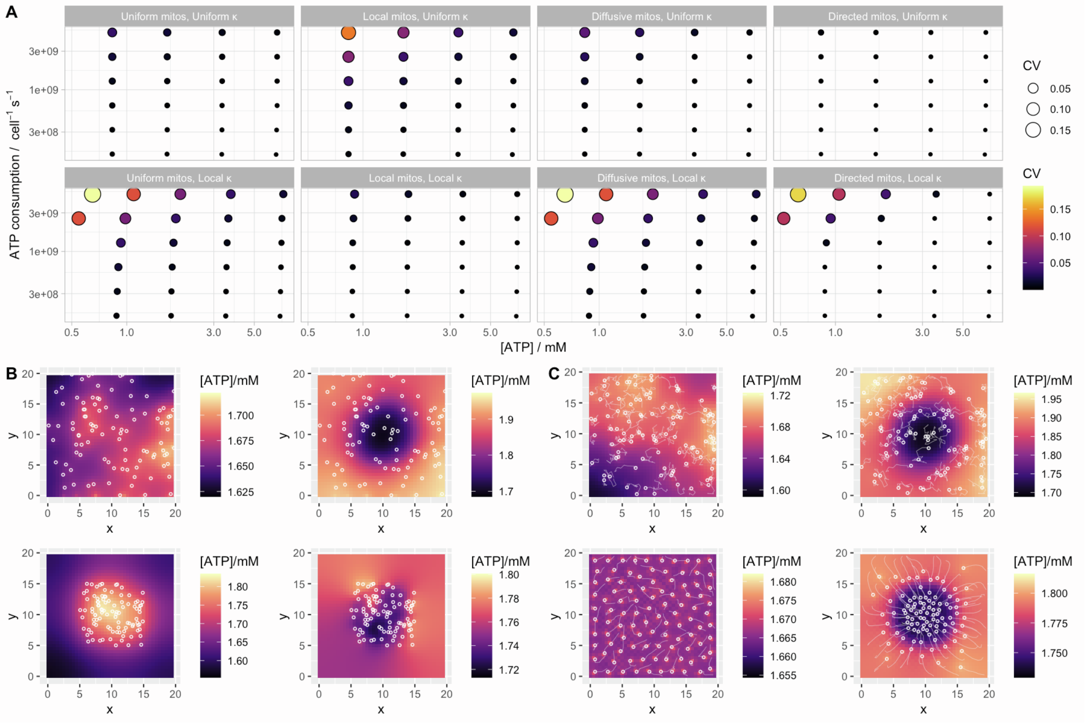

Supplementary Figure 2. **Smaller cell version of the model.** This approach uses a model cell 20 μm × 20 μm × 10 μm (hence volume 4 × 10^3 μm^3). (A) CV of ATP concentration as in Fig. 3. (B) Long-term concentration profile with static mitochondria as in Fig. 2. (C) Long-term concentration profile with static mitochondria as in Fig. 4. Parameters in (B-C) correspond to cellular consumption rates around 2.6 × 10^9 molecules s^{-1}.

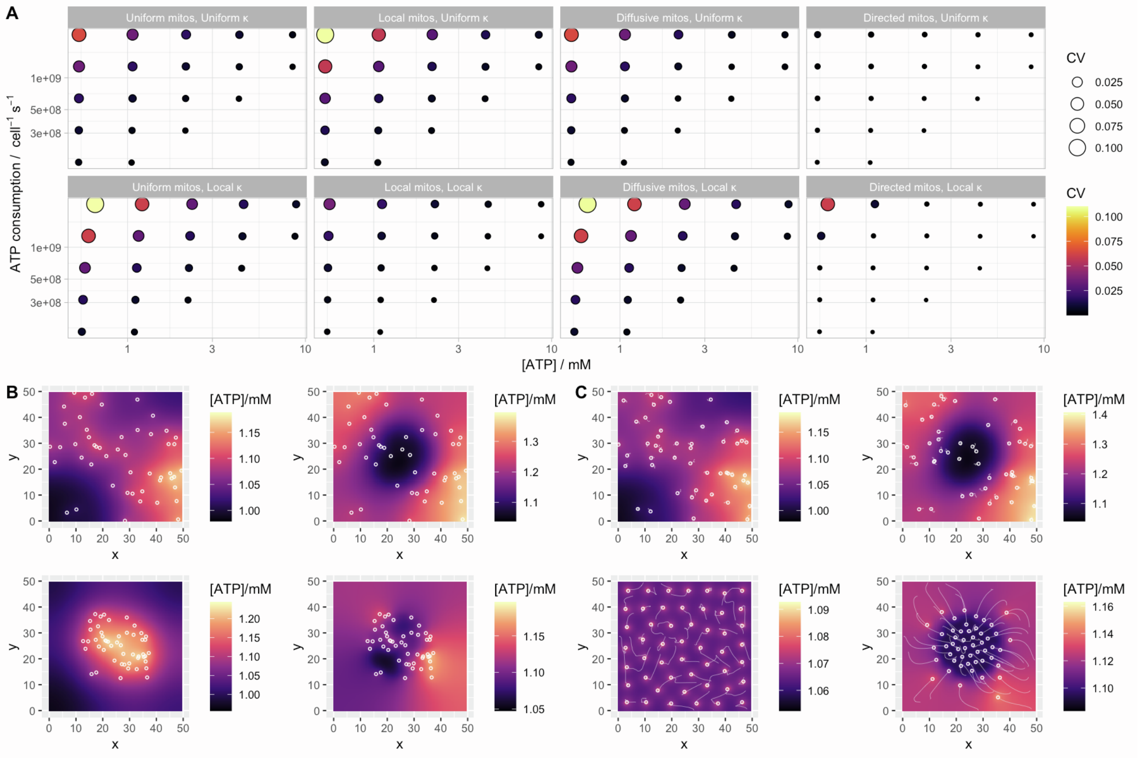

Supplementary Figure 3. **Model with fewer mitochondria.** 50 mitochondria in the simulated cell. (A) CV of ATP concentration as in Fig. 3. (B) Long-term concentration profile with static mitochondria as in Fig. 2. (C) Long-term concentration profile with static mitochondria as in Fig. 4.

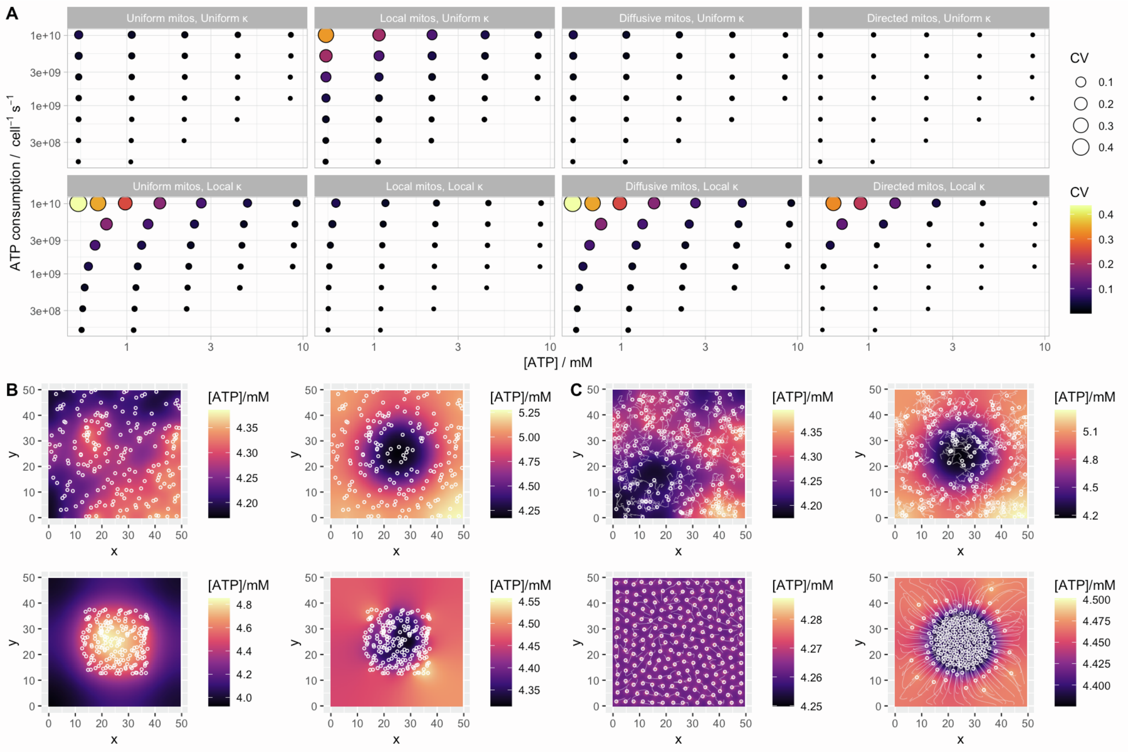

Supplementary Figure 4. **Model with more mitochondria.** 200 mitochondria in the simulated cell. (A) CV of ATP concentration as in Fig. 3. (B) Long-term concentration profile with static mitochondria as in Fig. 2. (C) Long-term concentration profile with static mitochondria as in Fig. 4.

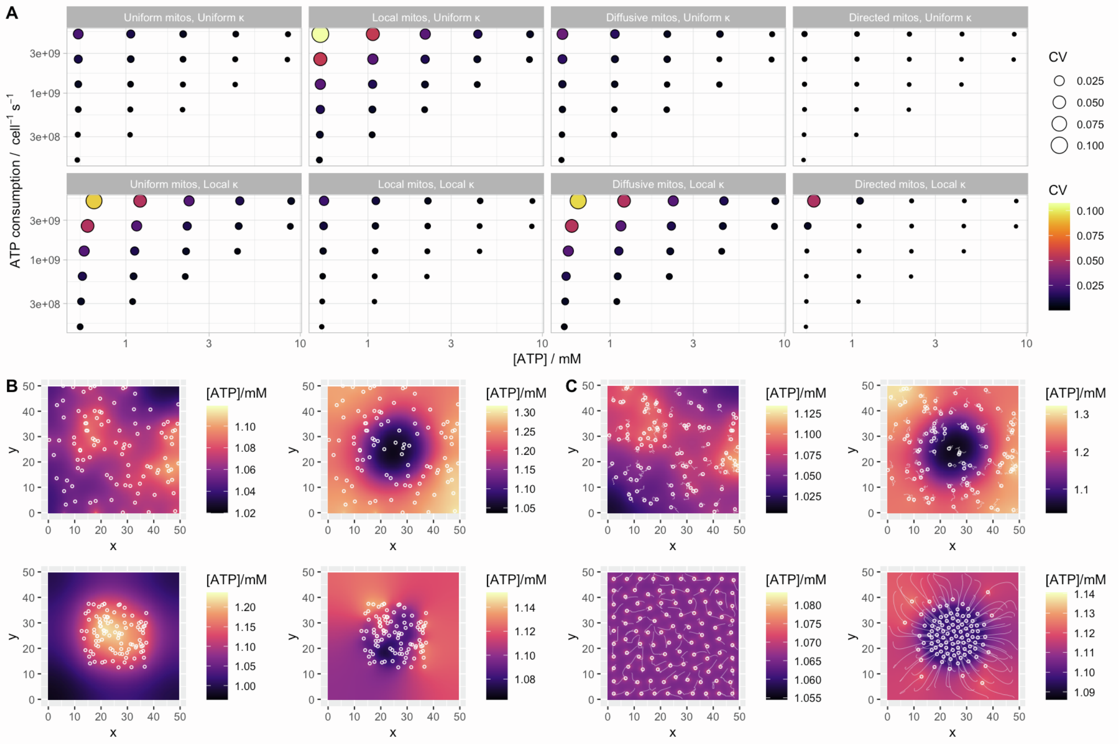

Supplementary Figure 5. **Model with higher cell thickness.** Cell thickness assumed to be 20µm. (A) CV of ATP concentration as in Fig. 3. (B) Long-term concentration profile with static mitochondria as in Fig. 2. (C) Long-term concentration profile with static mitochondria as in Fig. 4.

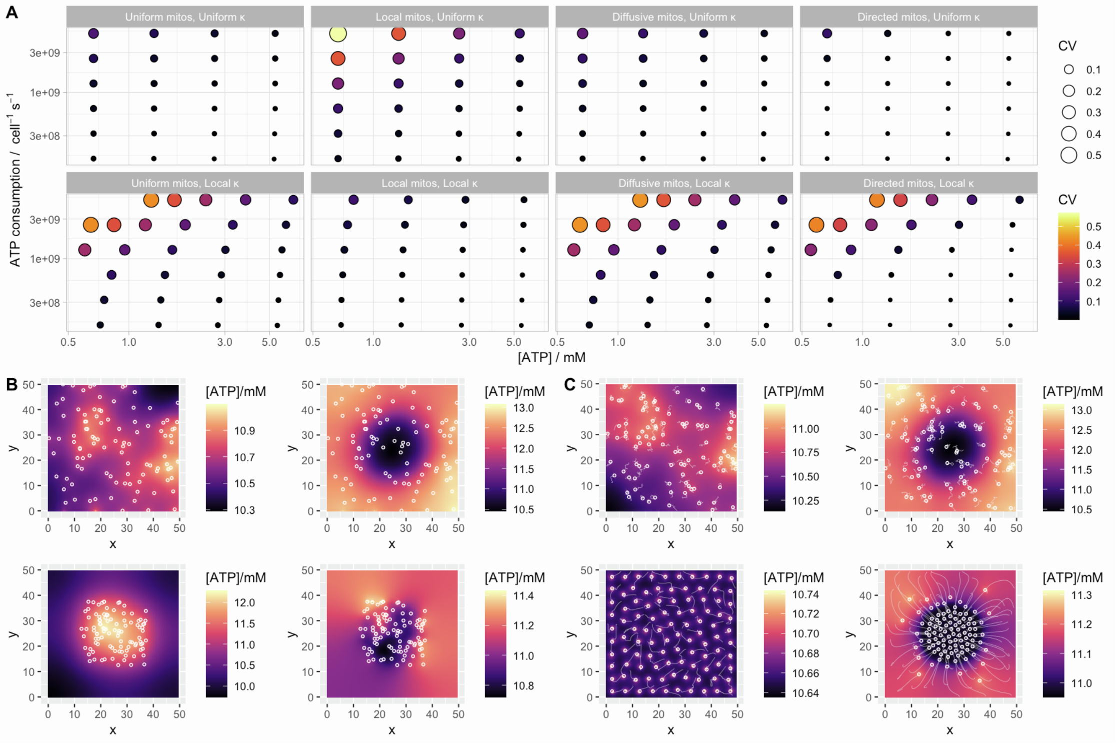

Supplementary Figure 6. **Model with lower cell thickness.** Cell thickness assumed to be 2µm. (A) CV of ATP concentration as in Fig. 3. (B) Long-term concentration profile with static mitochondria as in Fig. 2. (C) Long-term concentration profile with static mitochondria as in Fig. 4.
